# Small Molecule Palmatine Targeting Musashi-2 in Colorectal Cancer

**DOI:** 10.3389/fphar.2021.793449

**Published:** 2022-01-27

**Authors:** Xue Zhang, Kaiyan Su, Yifan Liu, Darong Zhu, Yuting Pan, Xisong Ke, Yi Qu

**Affiliations:** ^1^ Shanghai Frontiers Science Center for Chemical Biology, Institute of Interdisciplinary Integrative Medicine Research, Shanghai University of Traditional Chinese Medicine, Shanghai, China; ^2^ Department of Pharmacy, Shanghai Jiao Tong University Affiliated Sixth People’s Hospital, Shanghai, China

**Keywords:** musashi-2, RNA-binding protein, colorectal cancer, palmatine, musashi-2 targets

## Abstract

Musashi-2 (MSI2) is an evolutionally conserved RNA-binding protein and recently considered as an attractive therapeutic target in a wide spectrum of malignancies. However, MSI2-engaged mRNAs are not well profiled, and no MSI2-dependent antagonist is available so far. In the study, we created MSI2 knockout cancer cells and demonstrated that MSI2 is required for the survival of colorectal cancer HCT116 cells but not non-small cell lung cancer A549 cells. In addition, the global profiling of the transcriptome and proteomics of MSI2 knockout colorectal cells revealed 38 candidate MSI2-targeted genes. In a loss–rescue screening, palmatine was identified as a functional MSI2 antagonist inhibiting the MSI2-dependent growth of colorectal cancer cells. Finally, we confirmed that palmatine is directly bound to MSI2 at its C-terminal. Our findings not only indicated MSI2 as a promising therapeutic target of colorectal cancer but also provided a small molecule palmatine as a direct and functional MSI2 antagonist for cancer therapy.

## Introduction

Musashi (MSI) is an evolutionally conserved RNA-binding protein (RBP) that was initially connected with asymmetric stem cell division and cell fate determination in the neuroblast (Nakamura et al., 1994). In addition to the fundamental requirement for controlling RNA fate, it has been recognized recently that the mammalian MSI family proteins (MSI1 and MSI2) are potential drivers of a range of malignancies (Hope et al., 2010; Kharas et al., 2010; Park et al., 2014). In particular, MSI2 is ubiquitously elevated and proposed as a novel biomarker and therapeutic target of hematopoietic malignancies and colorectal cancers (Li et al., 2015).

As an RBP, the oncogenic role of MSI2 in tumorigenesis is due to the dysregulation of its RNA targets (Schuschel et al., 2020). The identification of RNA targets in the cancer contexts allows to clarify the molecular function of MSI2 that is essential to define the MSI2-dependent carcinogenesis and the targeted therapeutic agents. Previous studies have identified direct MSI2 mRNA targets using cross-linking and immunoprecipitation technology in acute myeloid leukemia and leukemic stem cells (Vu et al., 2017). However, most of the studies were limited to hematopoiesis, and the step of cross-linking potentially led to off-targets (de Andrés-Aguayo et al., 2012; Park et al., 2014). A novel concise and reliable strategy is required to profile the specific MSI2-targeted mRNA in solid tumors.

As an RBP, there are challenges to develop small-molecule targeting MSI2 due to the lack of a typical deep hydrophobic pocket. Previous studies reported two small molecules Ro 08-2750 and (-)gossypol targeting MSI2 in colon cancer cells (Clingman et al., 2014; Lan et al., 2018; Minuesa et al., 2019); however, no small molecule has been validated for the requirement of MSI2 for its anti-cancer activities, especially for gossypol that was identified as an inhibitor of oncoprotein BCL2 previously (Zeng et al., 2019). Considering the golden standard in defining a drug’s putative target is that the depletion of a target confers resistance to the drug (Kapoor and Miller, 2017); it is essential to identify small molecules specifically sensitive to the wild-type (WT) but not MSI2-depleted cancer cells. In the study, we aim to identify MSI2 RNA targets and small molecules inhibiting MSI2-dependent growth in cancer cells and developed novel strategies based on the knockout of MSI2.

## Materials and Methods

### Chemicals and Reagents

A natural product library was provided by the School of Pharmacy, Second Military Medical University (Shanghai, China). Betulonic acid (HY-N1451), arctiin (HY-N0034), and palmatine chloride (HY-N0110) were purchased from MedChemExpress. Shizukaol F was a gift from the Second Military Medical University, Shanghai, P.R. China. Gossypol (N2135) and Ro 08–2750 (B6996) were purchased from APExBIO. The above compounds were all prepared as 10 mM stocks in DMSO and aliquots stored at −20°C and then further diluted in a phosphate-buffered solution (PBS) to the desirable concentration. The MSI2 rabbit monoclonal antibody (ab76148) was purchased from Abcam. The β-actin rabbit monoclonal antibody (8457) was obtained from Cell Signaling Technology. α-Tubulin monoclonal antibody (66031-1-Ig) were purchased from Proteintech. Peroxidase-AffiniPure Goat Anti-Rabbit IgG (H+L) (111-035-003) and anti-Mouse IgG (H+L) (115-035-003) were obtained from The Jackson Laboratory. The IgG rabbit antibody (I5006) was purchased from Sigma.

### Cell Culture

HCT116 (CCL-247) cells were obtained from the American Type Culture Collection. The HEK293FT cell line (R70007) was purchased from Thermo Fisher Scientific. The A549 cell (SCSP-503) was purchased from the National Collection of Authenticated Cell Cultures. The RPMI1640 medium for HCT116 and DMEM for HEK293T were purchased from Gibco. All cultures were supplemented with 10% FBS (Gemini) and penicillin-streptomycin (100 U/ml and 100 μg/ml, respectively; Gibco) and grown at 37°C in a 5% CO_2_-humidified chamber. The identity for all human cell lines in this study was confirmed by short tandem repeat (STR) analysis.

### CRISPR/Cas9-Mediated Genomic DNA Deletion

The whole protein-coding region of MSI2 was deleted in HCT116 and A549 cells using the CRISPR/Cas9 system. Two single-guide RNAs (sgRNA1 and sgRNA2, Table 1) were designed to flank the fragment to be deleted. sgRNA oligonucleotides were cloned in the pL-CRISPR.EFS.GFP vector (Addgene 57818) using BsmBI restriction digestion. The two sgRNA (sgRNA1 and sgRNA2) constructs were equally mixed and transfected into cells with lipofectamine 3,000 (Invitrogen L3000015) for 2 days to allow sufficient gene editing; the GFP-positive cells were purified using a four-laser cell sorter (MoFlo XDP, Beckman Coulter, Brea, CA, United States) and plated in 96-well plates at the density of one cell per well. To do a screen deletion of clones, single cell-derived colonies in 96-well plates were divided into two wells for PCR and passaging, respectively. Genomic DNA was extracted from each clone, followed by PCR amplification flanking the cut site screening using primers detecting external (Ext) and internal (Int) regions (listed in Table 1), respectively. The temperature steps for both primers were 95°C for 1 min, which was followed by 30 cycles of 95°C for 30 s, 60°C for 30 s, and 72°C for 30 s, with a final primer extension step at 72°C for 10 min. The PCR product was sequenced with the corresponding forward primer, and positive clones were further confirmed by Western blot.

**TABLE 1:** sgRNA and primers sequence used in CRISPR/Cas9.

Oligo	Sequence (5′ to 3′)
sgRNA1 (FWD)	CAC​CGG​AGT​CGT​TGG​CGC​TGC​CCG
sgRNA1 (RED)	AAA​CCG​GGC​AGC​GCC​AAC​GAC​TCC
sgRNA2 (FWD)	CAC​CGA​GTG​GGG​AAT​TAC​ATA​AGT​G
sgRNA2 (RED)	AAA​CCA​CTT​ATG​TAA​TTC​CCC​ACT​C
MSI2 Ext (FWD)	ATGGAGGCAAATGGGAGC
MSI2 Ext (RED)	CAG​GGA​AAT​GAG​ATT​CAG​ACG
MSI2 Int (FWD)	GAT​CCC​ACT​ACG​AAA​CGC​TCC​A
MSI2 Int (RED)	GTC​TGC​GAA​CGT​GAC​GAA​ACC

### Cell Viability and Colony Formation Assay

The cell viability assay was determined using cell counting kit-8 (CCK8, APExBIO K1018) according to the manufacturer’s instructions. Normalized cell viability was calculated as a percentage of the DMSO control.

Cells were seeded at 750 cells/well in a 12-well plate, and the medium was changed each 3 days. Seven days later, the culture medium was discarded, colonies were washed with PBS for three times, fixed with 4% paraformaldehyde solution for 20 min and stained with 0.1% crystal violet (Beyotime Biotechnology C0121) for 10 min, and then washed and dried at room temperature.

### Western Blotting

Cells were lysed in ice-cold RIPA (9,806, Cell Signaling) with PMSF (A100754, Sangon Biotech) and protease inhibitors (A32953, Thermo Scientific) for 20–30 min, followed by centrifugation, quantification, and denaturation. Twenty micrograms protein from each sample was separated to 8% SDS-PAGE gels, then transferred onto PVDF membranes and blocked with 5% non-fat milk. Membranes were sequentially incubated with primary antibodies and HRP-labeled secondary antibodies. The signals were detected by chemiluminescence using the ChemiDoc MP Imaging System (Bio-Rad). The bands’ intensity was measured by the GelPro Analyzer software.

### Cell Cycle Analysis

Cells were collected and fixed with 70% ethanol at 4°C overnight. The cells were then centrifuged, washed by PBS, and stained using propidium iodide (PI)/RNase Staining Buffer (BD Pharmingen 550825) for 30 min at room temperature in the dark, followed by analysis by BD Accuri™ C6 Plus (BECKMAN COULTER CytoFLEX, CA, USA). At least 10,000 cells were used for each analysis, and the results were displayed as histograms.

### EdU Incorporation Assays

Cells were labeled with 10 μM EdU for 30 min and then stained using a BeyoClick™ EdU-488 Imaging Kit (Beyotime C0071S). Cellular nuclei were stained using Hoechst33342, and the images were acquired with the ImageXpress Micro 4 High-Content Imaging System.

### Quantitative Reverse Transcription-PCR Assays

Cells were collected and RNA was extracted by TRIzol (15596026, Thermo Scientific).

1 μg of total RNA was reverse-transcribed into cDNA according to the manufacturer’s protocols. Quantitative RT-PCR analysis was performed using the PowerUp™ SYBR™ Green Master Mix (A25742, Thermo Fisher Scientific) in the QuantStudio Q5 PCR machine (Thermo Fisher Scientific) with the specific primers of each gene (Table 2). β-actin was used as an internal reference gene to normalize mRNA levels. Data were analyzed with the 2^−ΔΔCt^ method. Each sample was tested in triplicate. The results were analyzed in CopyCaller software to determine the copy number of the target gene.

**TABLE 2:** Primers sequence used in qPCR.

Primers	Sequence (5′ to 3′)
Cyclin E1 (FWD)	GGA​GAT​GAA​ATT​CTC​ACC​ATG​G
Cyclin E1 (RED)	CAG​GAC​ACA​GAG​ATC​CAA​CAG
Cyclin D1 (FWD)	TGC​ATC​TAC​ACC​GAC​AAC​TCC
Cyclin D1 (RED)	CGT​GTT​TGC​GGA​TGA​TCT​GTT
β-actin (FWD)	CAT​GTA​CGT​TGC​TAT​CCA​GGC
β-actin (RED)	CTC​CTT​AAT​GTC​ACG​CAC​GAT

### Apoptosis Analysis

Cells were collected and washed with ice-cold PBS. The cells were resuspended with 1 × binding buffer to adjust the cell density as ∼1 × 10^6^ cells/ml and stained with 5 μl Alexa Fluor 488 annexin V and 1 μl 100 μg/ml PI working solution (V13241, Invitrogen) at room temperature in the dark for 15 min. After the incubation, a 400 μl 1 × annexin-binding buffer was added to the cells, mixed gently, and analyzed by flow cytometry.

### Xenograft Tumor Assay

All the animal experiments were carried out in accordance of the Institutional Animal Care and Use Committee of Shanghai University of Traditional Chinese Medicine (PZSHUTCM200807005). Five-week-old immunodeficient BABL/c male nude mice (20 ± 2 g) were purchased from Sino-British SIPPR/B&K Lab Animal Ltd., and maintained under specific pathogen-free conditions with constant temperature (23 ± 2°C) and controlled light (12 h light: 12 h dark).

After 1 week of acclimation, the colorectal cancer cells of HCT116 WT or MSI2 knockout cells (10^6^ in 0.2 ml) were injected into the right armpit of nude mice. Five days later, each nude mouse formed a palpable tumor. When the tumor volume reached about 50 mm^3^, nude mice with wide-type HCT116 cells were grouped randomly and intraperitoneally injected with PBS or palmatine (50 mg/kg/day) for 13 days. Tumor volumes were measured every other day and calculated using the formula: tumor volume (mm^3^) = (longer diameter × shorter diameter^2^)/2. At the end of experiment, mice were anesthetized with 4% chloral hydrate (Sigma) and sacrificed and tumors were excised, weighed, and measured.

### Proteome Profiling

Cells were lysed with a lysis buffer [50 mM ammonium bicarbonate, 8 M urea, and protease inhibitor (A32953, Thermo Scientific)] under ultrasound in ice for 30 min. After centrifugation (14,000 rpm, 15 min, 4°C), 5 mM dithiothreitol (DTT) at 37°C for 1 h, 10 mM iodoacetamide at room temperature for 45 min and 50 mM ammonium bicarbonate to diluted urea to 1 M were performed in sequence. The protein solution was then digested by trypsin in a ratio of 1:50 at 37°C overnight. Peptides were separated and analyzed on an Easy-nLC 1,200 system coupled to an Orbitrap Fusion (Thermo Fisher Scientific). Raw data were processed and searched with MaxQuant 1.5.4.1 with an MS tolerance of 5 ppm and an MS/MS tolerance of 20 ppm. The UniProt human protein database (release 2016_07, 70,630 sequences) and the database for proteomics contaminants from MaxQuant were used for database searches. Student’s *t* test was used to determine the statistical significance of protein abundances between test and control samples. A *p*-value <0.05 was considered to be statistically significant. A second threshold based on a fold change of greater than 1.5-fold or less than 1.5-fold was chosen so as to focus the data analysis on a small set of proteins with the largest alterations in abundance.

### RNA Sequencing and Data Processing

Total RNA was isolated using the RNeasy Mini Kit (74014, Qiagen) according to the manufacturer’s instruction. About 200 ng of total RNA was used to prepare the sequencing libraries using Illumina TruSeq Stranded Total RNA Sample Preparation Kit (Illumina) based on the manufacturer’s protocol. RNA sequencing was performed by BGI Genomics using BGISEQ-500 platform at Wuhan, China with 100-bp paired-end reads.

The high-quality sequencing reads were aligned to the human reference genome (hg38, UCSC) using the Burrows–Wheeler aligner (BWA, v0.7.15a). The gene expression level was measured by fragments per kilobase of transcript per million fragments (FPKM). Particularly, the gene expression in MSI2^−/−^ cells was defined as the average of that in the three (KO3, KO11, and KO21) or two (KO8 and KO9) MSI2 knockout HCT116 or A549 cells, respectively. The fold change of FPKM (MSI2^−/−^/WT) >2 or <0.5 and a *P*-value cutoff value <0.05 were applied to evaluate differentially expressed genes (DEGs). A total of 1,045 and 598 downregulated DEGs were identified in HCT116 and A549 cells for Kyoto Encyclopedia of Genes and Genomes (KEGG) analyses that were executed by the enrichKEGG functions of the clusterProfiler package, respectively (H. Li and Durbin, 2010). with the DEGs with *p* < 0.05 used as the threshold to select KEGG terms. For gene set enrichment analysis (GSEA), all the genes were inputted and analyzed using the GSEA function of clusterProfiler package (H. Li and Durbin, 2010) with hallmark gene sets (h. all. v7.1). For volcano plots, all the genes were input and analyzed using the ggplot2 package of the R language.

### RNA Immunoprecipitation-qPCR

Cells were harvested and washed twice with ice-cold PBS, followed by resuspension in an equal pellet volume of a polysome lysis buffer [100 mM KCl, 5 mM MgCl_2_, 10 mM HEPES pH 7.0, 0.5% Nonidet P-40 (NP-40), 1 mM DTT, 200 units/ml RNase OUT, and an EDTA-free Protease Inhibitor Cocktail]. The cell lysate was incubated with Protein G beads (10004D, Thermo Scientific) which were pre-bound with MSI2 or an IgG antibody overnight at 4°C, followed by washing with an NT-2 buffer (50 mM Tris pH 7.4, 150 mM NaCl, 1 mM MgCl_2_ and 0.05% NP-40) and resuspended with SDS-proteinase K (NT-2 buffer supplemented with 1% SDS and 1.2 mg/ml Proteinase K). RNA was extracted from the beads complex by phenol: chloroform: isoamyl alcohol (25:24:1) and precipitated by precipitation buffer [50 μl of ammonium acetate 5 M, 15 μl of LiCl 7.5 M, 5 μl glycogen (5 mg/ml), and 850 μl of absolute ethanol]. The pellet was resuspended in an RNase-free TE buffer. Immunoprecipitated RNA was subjected to quantitative PCR (qPCR) analysis as described above with the specific primers of each gene (Table 3).

**TABLE 3:** Primers sequence used in RIP-qPCR.

Primers	Sequence (5′ to 3′)
KIF18A (FWD)	TGC​TGG​GAA​GAC​CCA​CAC​TAT
KIF18A (RED)	GCT​GGT​GTA​AAG​TAA​GTC​CAT​GA
ZFHX4 (FWD)	CGG​TCA​TCT​GCT​GTC​CTC​T
ZFHX4 (RED)	CAC​ATT​AGC​CTT​TGA​ATC​CTC
PEG10 (FWD)	AGC​AGT​CGG​AGG​AGA​ACA​AC
PEG10 (RED)	CAC​TGG​GCC​ATG​AAA​GGA​G
EIF4EBP1 (FWD)	GCA​ATA​GCC​CAG​AAG​ATA​AGC​G
EIF4EBP1 (RED)	TCA​GTG​GAG​GCA​CAA​GGA​GGT

### MSI2-Dependent Inhibitors Screening

HCT116 WT and knockout cells (4 * 10^4^ cells/well) were plated in 96-well plates overnight followed by treatment with compounds (10 μM) in the library for 72 h. Compounds in the library were stocked in 100% DMSO at a concentration of 10 mM; therefore, 10% DMSO in PBS was controlled as the control in the screening. The CCK8 assay was used to evaluate the cell viability, and compounds with more than three-fold viability in the MSI2 knockout cells than the WT were selected for further confirmation.

### Microscale Thermophoresis Binding Assay

The microscale thermoporesis (MST)-binding assay using NanoTemper monolith NT.115 (NanoTemper Technologies GmbH). Briefly, the HEK293FT cell was overexpressed with the GFP-tagged protein of interest for 24 h, followed by preparing the cell lysates. The compound was serially diluted in an MST buffer (50 mM Tris-HCl buffer, pH 7.6, 150 mM NaCl, 10 mM MgCl2, and 0.05% Tween-20) and then incubated with cell lysates for 3–5 min. The sample was loaded into Monolith NT standard capillaries (MO-K022, NanoTemper Technologies), and the MST measurements were performed at 25°C, 40% LED power and 10% IR-laser power. Kd values were calculated using the mass action equation available in NanoTemper software.

### Cellular Thermal Shift Assay

Cells were incubated with DMSO (0.1% v/v) or palmatine (100 μM) for 1 h before they were collected and equally divided into seven tubes. Each tube was incubated at a specific temperature for 3 min and then left it at room temperature for 3 min. After heating, the cells were subjected to liquid nitrogen and 25°C freeze–thaw cycles for three times. The cell lysates were centrifuged at 14,000 g for 15 min at 4°C, and the supernatants were transferred for immunoblotting analysis.

## Results

### MSI2 Is a Potential Therapeutic Target of Colon Cancer

**FIGURE 1:**
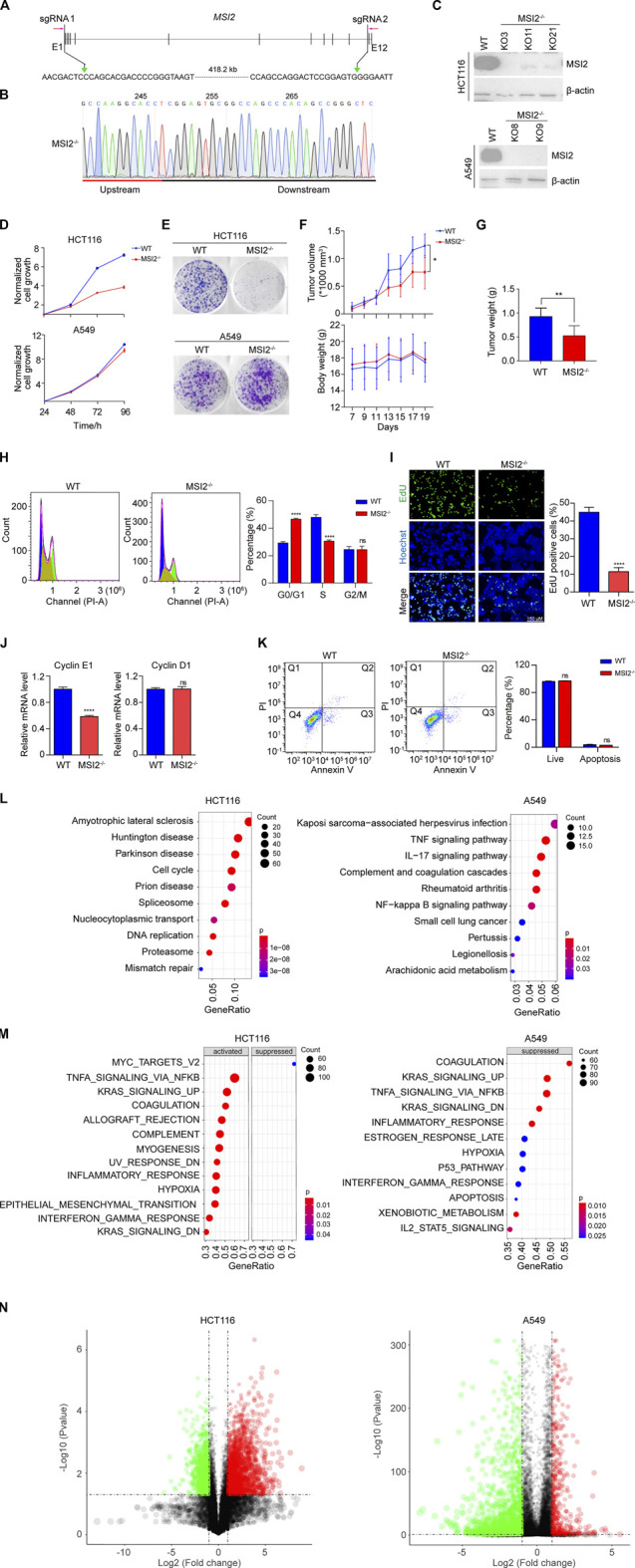
MSI2 is a potential therapeutic target of colon cancer. **(A)** Schematic depiction of CRISPR/Cas9-based genomic DNA deletion and PCR-based colony screening. Two sgRNAs were designed to target the coding sequence of MSI2. To screen MSI2-deleted colonies, one primer pair was designed, targeting the external deletion region (red arrows). **(B)** Genomic DNA sequencing confirmed the expected deletion at the sgRNA recognition sites in cancer cells containing GFP-tagged Cas9 and sgRNAs. **(C)** Immunoblotting analysis of MSI2 knockout HCT116 (KO3, KO11, and KO21) and A549 (KO8 and KO11) clones. **(D)** CCK8 assay of the viability of wild-type (WT) and MSI2 knockout (MSI2^−/−^) HCT116 and A549 cells cultured for the indicated time. Data of WT cells were indicated in blue, while the MSI2^−/−^’s were in red. Data were presented as mean ± SD of the three independent experiments. **©** Representative images of colony formation of WT and MSI2^−/−^ HCT116 and A549 cells cultured for 8 days. **(F)** Growth curves of xenograft tumors and body weight of WT or MSI2^−/−^ HCT116 xenograft nude mice. Data were presented as mean ± SD of six mice in each group, *p* < 0.05, two-way ANOVA. **(G)** Quantifications of MSI2 knockout HCT116 xenograft tumors’ weight. Data were presented as mean ± SD of six mice in each group, *p* < 0.01, unpaired *t*-test (two tailed). **(H)** Cell cycle assay was performed by staining of cells with propidium iodide (PI), followed by flow cytometry analysis. The scatter plots represented PI (*X*-axis) vs. counted cells (*Y*-axis). Cell cycle proportion was graphed at the right panel. **(I)** Fluorescent images and quantification of EdU incorporation in WT and MSI2^−/−^ HCT116 cells. Scale bar: 150 μM. **(J)** Quantitative PCR analysis of the mRNA expression of Cyclin E1 and Cyclin D1 in WT and MSI2^−/−^ HCT116 cells. **(K)** Representative scatter plots and quantification of apoptosis by Annexin V-FITC and PI staining in WT and MSI2^−/−^ HCT116 cells. Q1 (Annexin V^+^, PI^+^): dead cells; Q2 (Annexin V^−^, PI^+^): late-stage apoptosis cells; Q3 (Annexin V^+^, PI^−^): early-stage apoptosis cells; Q4 (Annexin V^−^, PI^−^): live cells. Data were presented as mean ± SD of three independent experiments. *p* < 0.01, *p* < 0.001, *p* < 0.0001, ns: not significant, two-way ANOVA **(H**–**K)** and unpaired *t*-test **(I**,**J)**. MSI2 is a potential therapeutic target of colon cancer. **(L)** Dot plotting of the top 10 pathways significantly enriched by downregulated genes in MSI2 knockout HCT116 and A549 cells. DEGs were identified as fold change (MSI2^−/−^/WT) <0.5 and *alue*aue <0.05, where the mRNA level of MSI2^−/−^ cells were defined as the average of that in the three (KO3, KO11, and KO21) or two (KO8 and KO9) MSI2 knockout HCT116 or A549 cells, respectively. *Y*-axis represents pathways; *X*-axis represents the ratio of the MSI2-related mRNAs enriched in KEGG pathways. The color and size of each bubble represent enrichment significance and the number of MSI2-related mRNAs enriched in a pathway, respectively. **(M)** Global and unbiased gene expression pattern analysis (GSEA) of MSI2^−/−^ cells using the hallmark gene set from MSigDB. The mRNA level of MSI2^−/−^ cells was the average of three (KO3, KO11, and KO21) or two (KO8 and KO9) MSI2 knockout HCT116 or A549 cells, respectively. *Y*-axis represents hallmark biological states or processes. *X*-axis represents the enriched genes’ ratio in the specific hallmark pathways. The color and size of each bubble represent enrichment significance and gene numbers enriched in hallmark pathways, respectively. **(N)** Volcano plots depicting the differential genes expression between wild-type and MSI2^−/−^ cells. Red dot represents significantly upregulated mRNA (log_2_ fold change >1, *p*-value < 0.05), Green dot represents significantly downregulated metabolite (log_2_ fold change ≤1, *p*-value < 0.05). Fold change is calculated as MSI2^−/−^/WT.

To validate the oncogenic role of MSI2 in colorectal cancer, MSI2 was knocked out in colorectal cancer HCT116 cells using CRISPR/Cas9 technology (Figure 1A). The genetic deletion of the MSI2 gene and the protein depletion of MSI2 were confirmed by DNA sequencing and Western blot, respectively (Figures 1B,C). Consistent with the previous findings, MSI2 knockout cells significantly inhibited cell growth in the assays of cell viability and colony formation (Figures 1D,E). The requirement of MSI2 for the survival of colorectal cancer HCT116 cells was also confirmed in xenograft assays, in that the MSI2 knockout tumors grew significantly slower than the WT ones, although the tumors initiated simultaneously (Figure 1F), and no obvious change of the body weight was observed in the WT and MSI2 knockout HCT116 xenograft mice (Figure 1F). Twenty days after xenografting, tumor weight was significantly reduced in the MSI2 knockout tumors (Figure 1G). Notably, in non-small cell lung cancer (NSCLC) A549 cells that MSI2 was depleted in parallel (Figure 1C), no significant change of the survival and proliferation of A549 cells was detected upon depletion of MSI2 (Figures 1D,E). Actually, the oncogenic role of MSI2 in colon cancer was supported by an independent report using the knockdown assay (Li et al., 2015); more NSCLC cell lines are needed to distinguish the role of MSI2 in colon and lung cancers.

To explore the underlying mechanism for growth inhibition in MSI2-depleted HCT116 cells, the cell cycle and apoptosis examination were performed. Notably, the loss of MSI2 significantly increased the proportion of arrested cells at G0/G1 and decreased the number of S-phase cells (Figure 1H), which was supported by EdU incorporation experiments that the proportion of S-phase cells was significantly reduced in MSI2 knockout cells (Figure 1I). The critical regulators of G1-to-S phase transition were examined using qPCR, in that the transcription of Cyclin E1 was significantly decreased in the cells’ absence of MSI2, while the cyclin D1 mRNA level was not changed (Figure 1J). Moreover, the depletion of MSI2 did not affect the apoptosis of HCT116 cells compared with the WT (Figure 1K). Collectively, these results demonstrated that the depletion of MSI2 delayed the G1-to-S phase transition by regulating the amount of Cyclin E1 expression in colon cancer HCT116 cells, thus suppressing its proliferation. To better understand the role of MSI2 in human colon cancer, we profiled the global gene expression of MSI2 knockout HCT116 cells using RNA sequencing. As expected, the pathways associated with the RNA process such as spliceosome and nucleocytoplasmic transport were markedly downregulated in MSI2 knockout cells (Figure 1L). Notably, MSI2 depletion was significantly connected with neurological diseases, including amyotrophic lateral sclerosis, Huntington’s disease, Parkinson’s disease, and Prion’s disease, highlighting the critical role of MSI2 in the neuron system (Figure 1L), which is consistent to a previous study in that the pathological accumulation of MSI2 aggregates contributes to neurodegenerative proteinopathies (Montalbano et al., 2020). Additionally, a global and unbiased gene expression pattern analysis (GSEA) revealed that important cellular pathways such as Myc, TNFα-NFκB, and KRAS signaling were correlated significantly with the gene expression profile of MSI2 knockout HCT116 cells (Figure 1M).

A comparison analysis of the gene expression pattern was also performed in that of A549. Different from HCT116 cells, the KEGG analysis in A549 cells revealed a close correlation between the MSI2 and immune response, in that the loss of MSI2 significantly regulated infection; TNFα, IL-17, and NF-κB signal pathways; complement and coagulation cascades; and genes related to rheumatoid arthritis, a chronic autoimmune disease (Figure 1L), rather than its known function in RNA processing. In the GSEA analysis, most of the MSI2-affected biological functions in A549 cells overlapped with HCT116 cells, except the absence of the suppressed Myc pathway (Figure 1M). Since the central role of Myc is in the oncogenic process, this may partially explain the distinct proliferation pattern of HCT116 and A549 after the knockout of MSI2. Additionally, in depicting DEGs using volcano plots (Figure 1N), of interest was that most of the top changed genes (MSI2−/−/WT > 8 or < 0.125) were upregulated in HCT116 (51 of up-DEGs against 16 of down-DEGs), which is in contrast to A549 cells in that most DEGs were mainly downregulated (2 of up-DEGs against 38 of down-DEGs), and none of them were overlapped between two cell types. This observation could be potential explanation for the different roles of MSI2 in HCT116 and A549 cells as described (Figures 1L,M). Collectively, these data demonstrated that the loss of MSI2 in HCT116 was associated with decreased expression of proliferation and survival genes, while a unique association of MSI2 and immune response was identified in A549 cells.

### Identification of MSI2 RNA Targets in Human Colon Cancer Cells

**FIGURE 2:**
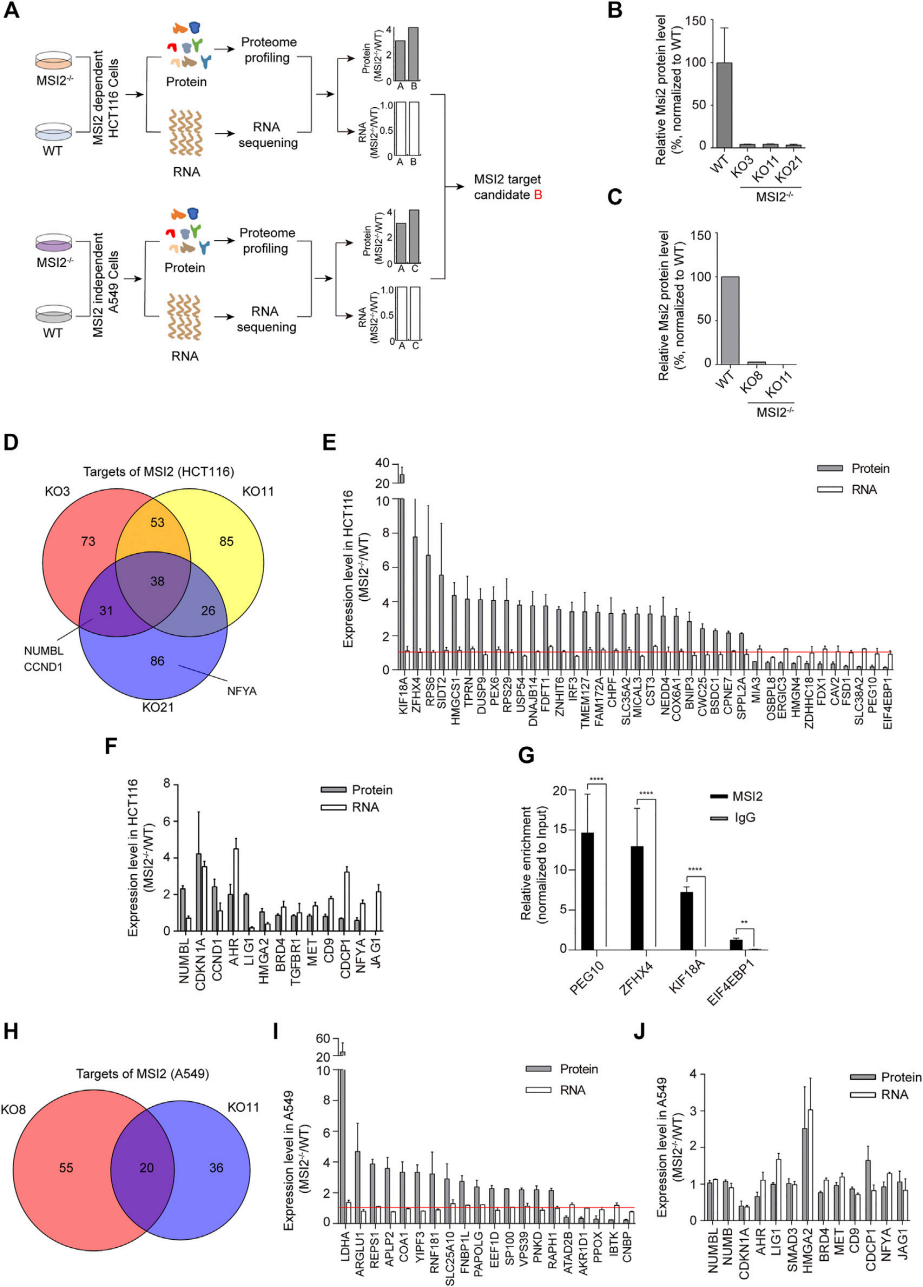
Identification of MSI2 RNA targets in human colon cancer cells. **(A)** Schematic depiction of strategy to identify the candidate targeted RNA of MSI2 specific in human colon cancer cells. “B” was indicated as the candidate target of MSI2 in HCT116 cells. **(B**,**C)** Mass spectrometry analysis of MSI2 protein level in MSI2 knockout clones of HCT116 cells **(B)** and A549 cells **(C)**. Data of B were presented as the mean ± SD of two independent experiments. **(D)** Venn diagram showing candidate targets of MSI2 overlapped between three MSI2 knockout HCT116 clones KO3 (red), KO11 (yellow), and KO21 (blue). **(E)** The ranking of the changed protein and RNA levels of MSI2-targeted candidates in MSI2^−/−^ HCT116 cells based on mass spectrometry and RNA sequencing, respectively. The red line indicated value “1.” **(F)** Ranking of the changed protein and RNA levels of known MSI2 targets in MSI2^−/−^ HCT116 cells. **(G)** RNA immunoprecipitation of MSI2 complexes with MSI2 or control rabbit IgG antibody, followed by qPCR detection of the selected MSI2 targets (KIF18A, ZFHX4, PEG10 and EIF4EBP1). Relative enrichment was calculated by enrichment over total input. Data were presented as mean ± SD of three independent experiments, *p* < 0.01, *p* < 0.0001, and one-way ANOVA. **(H)** Venn diagram showing candidate targets of MSI2 overlapped between the two MSI2 knockout A549 clones KO8 (red) and KO11 (blue). **(I)** Ranking of the changed protein and RNA levels of MSI2 targeted candidates in two MSI2^−/−^ A549 clones based on mass spectrometry and RNA sequencing. **(J)** Ranking of the changed protein and RNA levels of known MSI2 targets in MSI2^−/−^ A549 cells. Data of **(E**,**F)** were presented as mean ± SD of the three independent HCT116 knockout clones, and data of **(I**,**J)** were presented as mean ± SD of the two independent A549 knockout clones.

MSI2 is an RBP that binds RNA targets, resulting in translational alteration without affecting the overall mRNA stability. Therefore, candidate RNA targets can be determined according the different change patterns in mRNA and protein levels upon MSI2 depletion (Figure 2A). To identify MSI2-binding RNA targets in colon cancer cells, we took advantage of the MSI2 knockout cells and investigated the global changes of mRNA and protein by a combination of mass spectrometry and RNA sequencing, and the knockout and WT A549 cells were included as a control in parallel (Figure 2A). As expected, proteomic profiling revealed a striking depletion of MSI2 in both MSI2 knockout HCT116 and A549 cells (Figures 2B,C).

We analyzed the potential targets of MSI2 in each individual clone after the depletion of MSI2. More than 50% candidate targets in each clone were overlapped with those in the other MSI2 knockout HCT116 clones (Figure 2D). Moreover, a total of 38 genes were shared in all of the three clones, with significant alterations of protein abundance and almost unchanged mRNA levels upon MSI2 depletion (Figures 2D,E). Consistent to previous study (Ito et al., 2010; García-Alegría et al., 2016; Troiano et al., 2019), NUMBL (a close homologue of NUMB) and cyclin D1 (CCND1) were also included to MSI2 candidate targets in both MSI2 knockout clones KO3 and KO21 (Figure 2D), supporting the reliability of our strategy in the study. Nevertheless, some reported MSI2 targets (Wang et al., 2015; Kudinov et al., 2016; Tsujino et al., 2019) exhibited unexpected change patterns after MSI2 depletion, for instance, targets (CDKN1A, AHR, and LIG1) with both changed protein and mRNA levels and genes (HMGA2, CD9, CDCP1, NFYA, and JAG1) with unaltered protein and increased mRNA level (Figure 2F). In addition, there are a panel of genes were not reported previously (e.g., KIF18A and EIF4EBP1); thus, MSI2 immunoprecipitation followed by qPCR was performed and confirmed that the top changed candidates in HCT116 cells (KIF18A, KIF18A, ZFHX4, PEG10, and EIF4EBP1) were enriched in MSI2 immunoprecipitates’ control to the IgG antibody (Figure 2G), confirming their association with MSI2 proteins and supporting our strategy in identifying MSI2-targeted mRNA.

To determine the MSI2 mRNA targets specific in colon cancer, we investigated the transcripts regulated by MSI2 in NSCLC A549 cells as control and found 20 candidate genes in total (Figures 2H,I). However, no gene was commonly detected in both HCT116 and A549 cells with MSI2 depletion. Notably, in contrast to HCT116 cells, nearly none of the reported MSI2 targets was changed in the translation expression in MSI2 knockout A549 cells (Figure 2J), implying that MSI2 is not required for the survival and growth of A549 cells.

### Discovery of Small Molecules Targeting MSI2-Dependent Growth in Colon Cancer Cells

**FIGURE 3:**
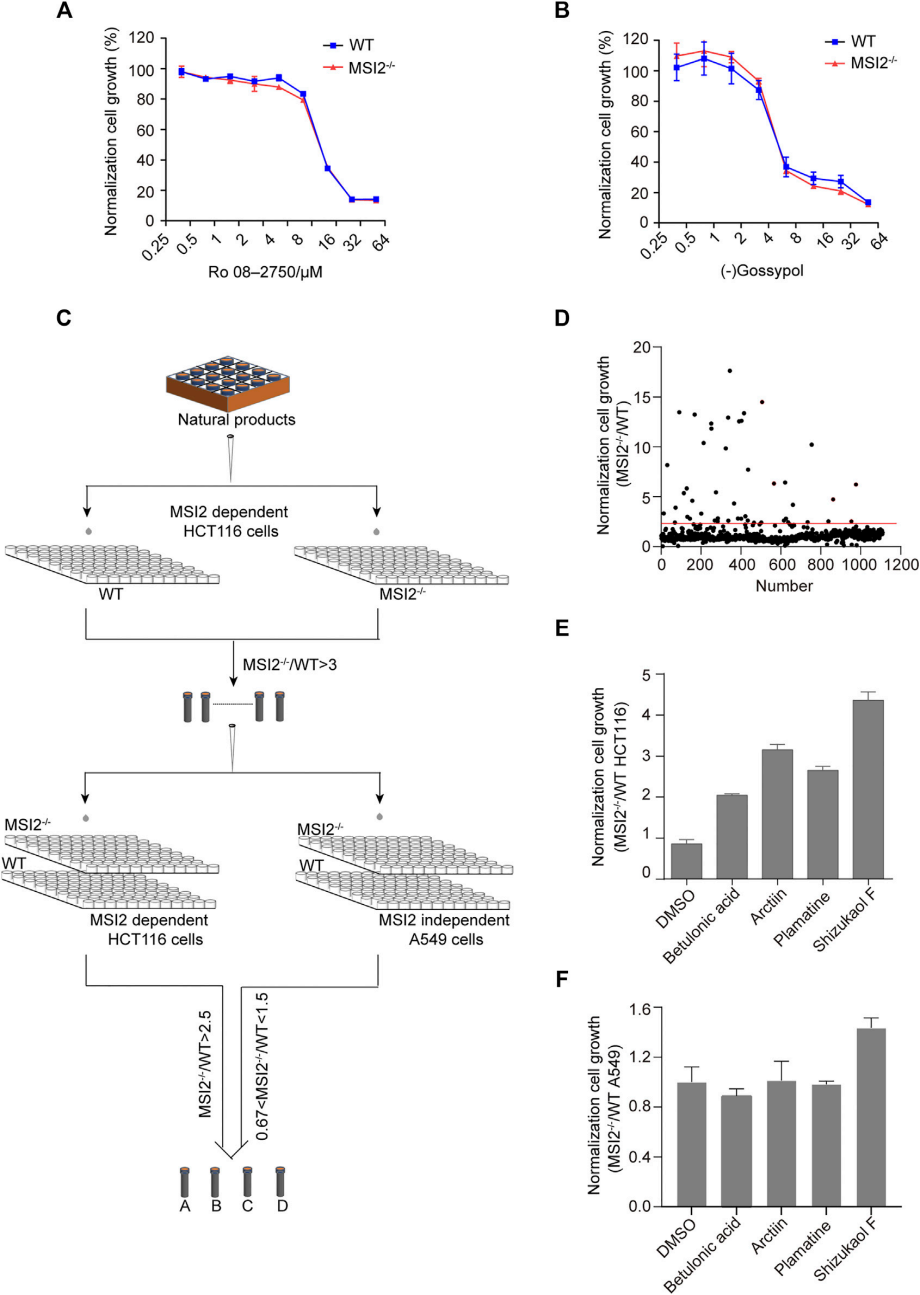
Discovery of small molecules targeting MSI2-dependent growth in colon cancer cells. **(A**,**B)** CCK8 assay of WT (blue) and MSI2^−/−^ (red) HCT116 cells upon treatment of Ro 08-2750 **(A)** or (-)Gossypol **(B)** for 72 h at indicated doses. Quantifications of three independent experiments were represented as mean ± SD. **(C)** Workflow of MSI2 inhibitor screening in MSI2-dependent colon cancer cells. Both WT and MSI2^−/−^ HCT116 cells were treated with a library of 1,109 natural compounds (10 μM) in parallel. Compounds with more than three-fold inhibition to WT compared to MSI2^−/−^ HCT116 cells in the first round of screening were selected for a next comparison in WT and MSI2^−/−^ cells in HCT116 paralleled to those of A549 cells. **(D)** Scatter plot showing the relative inhibition between WT and MSI2^−/−^ HCT116 cells in the first round of screening. Compounds for further confirmation were the dots above the red line. **(E**,**F)** CCK8 assay of WT and MSI2^−/−^ HCT116 **(E)** and A549 **(F)** cells upon treatment of betulonic acid, arctiin, shizukaol F, or palmatine at 10 μM for 72 h. Data were represented as mean ± SD of three independent experiments.

Previously, two small molecules Ro 08-2750 and (-)gossypol were identified as MSI2 antagonists *via* RNA binding competition assays (Clingman et al., 2014; Lan et al., 2018; Minuesa et al., 2019). To validate the functional blockade of MSI2 in colon cancer, both MSI2 knockout and WT HCT 116 cells were treated with Ro 08-2750 or (-)gossypol in parallel. Unexpectedly, no difference of cell growth inhibition was observed with either compounds to HCT116 cells regardless of MSI2 depletion, indicating that MSI2 was not required for the anti-cancer activities of either Ro 08-2750 or (-)gossypol in HCT116 cancer cells, although there are engagements between both compounds and MSI2 *in vitro* (Figures 3A,B).

To identify small molecule functionally targeting MSI2 in living cells, we performed a small molecule screening using MSI2 knockout and WT cells in parallel, and candidate inhibitors were defined by a selective inhibition to WT but not to MSI2 knockout HCT116 cells (Figure 3C). In the first round of screening using a library of 1,109 natural compounds, more than 20 compounds showed more than three-fold higher inhibition to WT compared with MSI2 knockout cells (Figure 3D). Four of them (betulonic acid, arctiin, shizukaol F, and palmatine) were confirmed in the following validation; all of them showed selective inhibition to WT HCT116 cells two times higher than MSI2 knockout cells (Figure 3E). As a control, no selectivity was found in WT and MSI2 knockout A549 cells treated with either compounds (Figure 3F), further supporting the functional targeting of MSI2 in colon cancer cells by these compounds.

### Palmatine Directly Engaged With MSI2 in the C-Terminal Domain

**FIGURE 4:**
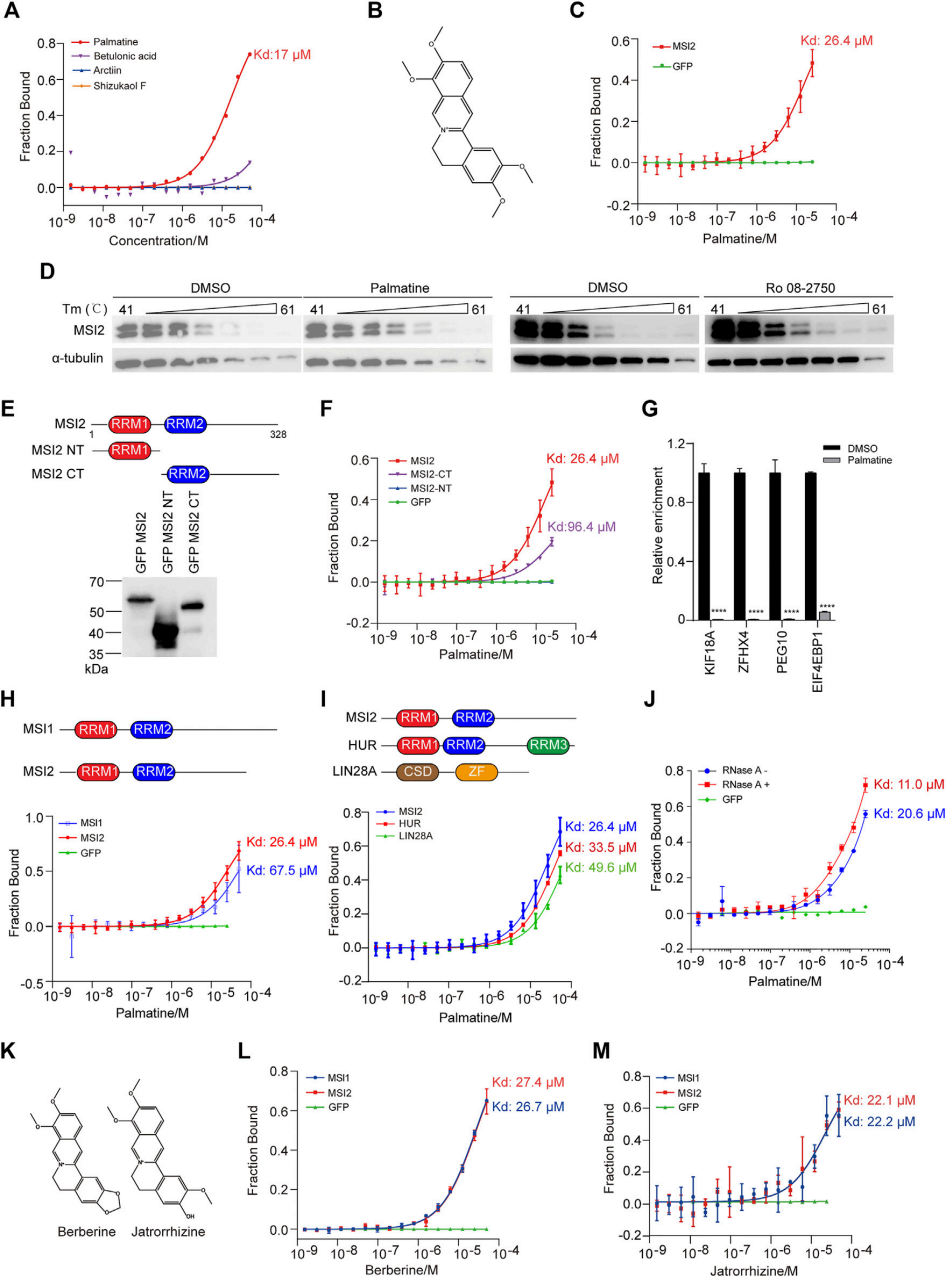
Palmatine directly engaged with MSI2 in the C-terminal domain. **(A)** MST analysis of the binding between MSI2 and the indicated compounds. **(B)** Chemical structure of palmatine. **(C)** MST assay of the binding between MSI2 and palmatine. GFP was used as a negative control. **(D)** CETSA assay of the engagement of MSI2 and palmatine in HCT116 cells. Cells were incubated with DMSO, palmatine (100 μM), or Ro 08-2750 (50 μM) at 37°C for 1 h before heating at indicated temperature. α-Tubulin was used as a loading control in Western blot analysis. **(E)** Schematic depiction of full length and truncations of MSI2 and Western blots of HEK293T cells’ overexpression with flag and GFP-tagged MSI2 or indicated truncations. **(F)** MST assay of palmatine binding to the indicated MSI2 truncations. GFP was used as a negative control. **(G)** RNA immunoprecipitation of MSI2 complexes in HCT116 cells treated with palmatine (100 μM) for 4 h qPCR detection of the selected MSI2 targets (KIF18A, ZFHX4, PEG10, and EIF4EBP1). Relative enrichment was calculated by enrichment over total input and then normalized to DMSO group. **(H)** Schematic depiction of MSI family proteins (MSI1 and MSI2) and MST assay of palmatine binding to the indicated MSI family proteins. GFP was used as a negative control. **(I)** Schematic depiction of RBPs (MSI2, HUR, and LIN28A) and MST assay of palmatine binding to the indicated RNA binding proteins. **(J)** MST assay of the binding between palmatine and MSI2. Blue and red indicated that cell lysate containing GFP-tagged MSI2 were treated with or without RNase A, respectively. Palmatine concentrations ranged from 0.00153 to 50 μM. Affinity (Kd) values ± SD (μM) of at least three independent experiments are shown as percentage of fraction bound. **(K)** Structure of berberine and jatrorrhizine. **(L**,**M)** MST assay of berberine **(L)** or jatrorrhizine **(M)** binding to the indicated MSI family protein. MST assays were performed as follows: HEK293FT was transfected with GFP-tagged gene, cell lysates was used to incubate with serially diluted compounds, and cell lysate containing GFP was used as a negative control. Data of **(C**,**F**,**G**,**I**,**K**,**L)** were presented as mean ± SD of the three independent experiments. Data were presented as mean ± SD of three independent experiments, *p* < 0.0001, two-way ANOVA.

To ask if the selective inhibition of four compounds to HCT116 cells was mediated by direct engagement with MSI2, we then examined the direct binding of the candidate compounds to MSI2 using the MST assay, an all-optical approach measuring the directed motion of the target protein in biological liquids (Wienken et al., 2010). Among the four compounds, a robust binding curve with a Kd at 17 μM was only detected when MSI2 was incubated with palmatine (Figures 4A,B), an active compound derived from the class of protoberberines (Long et al., 2019; Tarabasz and Kukula-Koch, 2020). As a control, the specific and direct binding of palmatine and MSI2 was confirmed using GFP-tagged protein in cell lysate (Figure 4C). To validate the engagement of palmatine and MSI2 in living cells, we further applied the cellular thermal shift assay (CETSA) in that the ligand engagement increases the thermal stability of the target protein against heating (Martinez Molina et al., 2013), and a significant thermal shift was observed with MSI2 in palmatine-treated cells (Figure 4D), further supporting the fact that MSI2 is a direct target of palmatine *in vitro* and *in vivo*. Similar to palmatine, CETSA showed a direct interaction between MSI2 and Ro 08-2750 (Figure 4D), which was in line with the previous study in that Ro 08-2750 bound MSI2 directly using MST (Minuesa et al., 2019).

As an RBP, MSI2 consists of two RRMs in the N-terminal (RRM1) and C-terminal (RRM2), which determines the RNA-binding specificity and binding affinity (Sakakibara et al., 2001). To figure out the binding sites of palmatine and MSI2, we created cells containing GFP-tagged MSI2, as well as the N-terminal truncation with RRM1 (NT) or C-terminal truncation with RRM2 (CT) (Figure 4E), and confirmed the correct expression in HEK293FT cells by Western blot (Figure 4E). In cellular protein-based MST assay, robust binding curve was detected when palmatine incubated with MSI2-CT but not MSI2-NT (Figure 4F), indicating that the C-terminal domain is essential for MSI2 binding to palmatine. However, the binding affinity of palmatine was lower with MSI2-CT (Kd = 96.4 μM) than the full-length protein (KD = 26.4 μM), indicating that the N-terminal is not sufficient but is required for MSI2 to achieve the highest binding affinity (Figure 4F). In line with this, palmatine significantly inhibited the RNA-binding affinity of MSI2 to its targets, such as KIF18A, ZFHX4, PEG10, and EIF4EBP1 (Figure 4G).

In addition, we examined the binding of palmatine to MSI1 containing homologous RRMs with similar binding specificities to target RNA (Figure 4G), MST analysis showed three times lower binding affinity of palmatine and MSI1 (Figure 4G), indicating the relative selectivity of palmatine binding to MSI2. We also performed MST assay of palmatine engagement with other RBPs including HUR and LIN28A and weaker-binding affinities were detected with HUR (33.5 μM) and LIN28A (49.6 μM), respectively (Figure 4I). Since alkaloids are known to bind RNA (Islam et al., 2009; Minuesa et al., 2019), to exclude RNA to palmatine/MSI2 interaction, RNase A was added to the protein samples before MST assay. Of interest, the binding affinity of palmatine and MSI2 was increased about two-fold in RNA-free samples (Figure 4J), supporting that palmatine directly interacts with MSI2 protein, and there could be a competition between palmatine and RNA. Moreover, similar binding affinities were also observed when berberine and jatrorrhirine (analogs of palmatine, Figure 4K) were incubated with MSI family proteins MSI1 and MSI2 (Figures 4L,M).

### palmatine Inhibited Colon Cancer *In Vitro* and *In Vivo*

**FIGURE 5:**
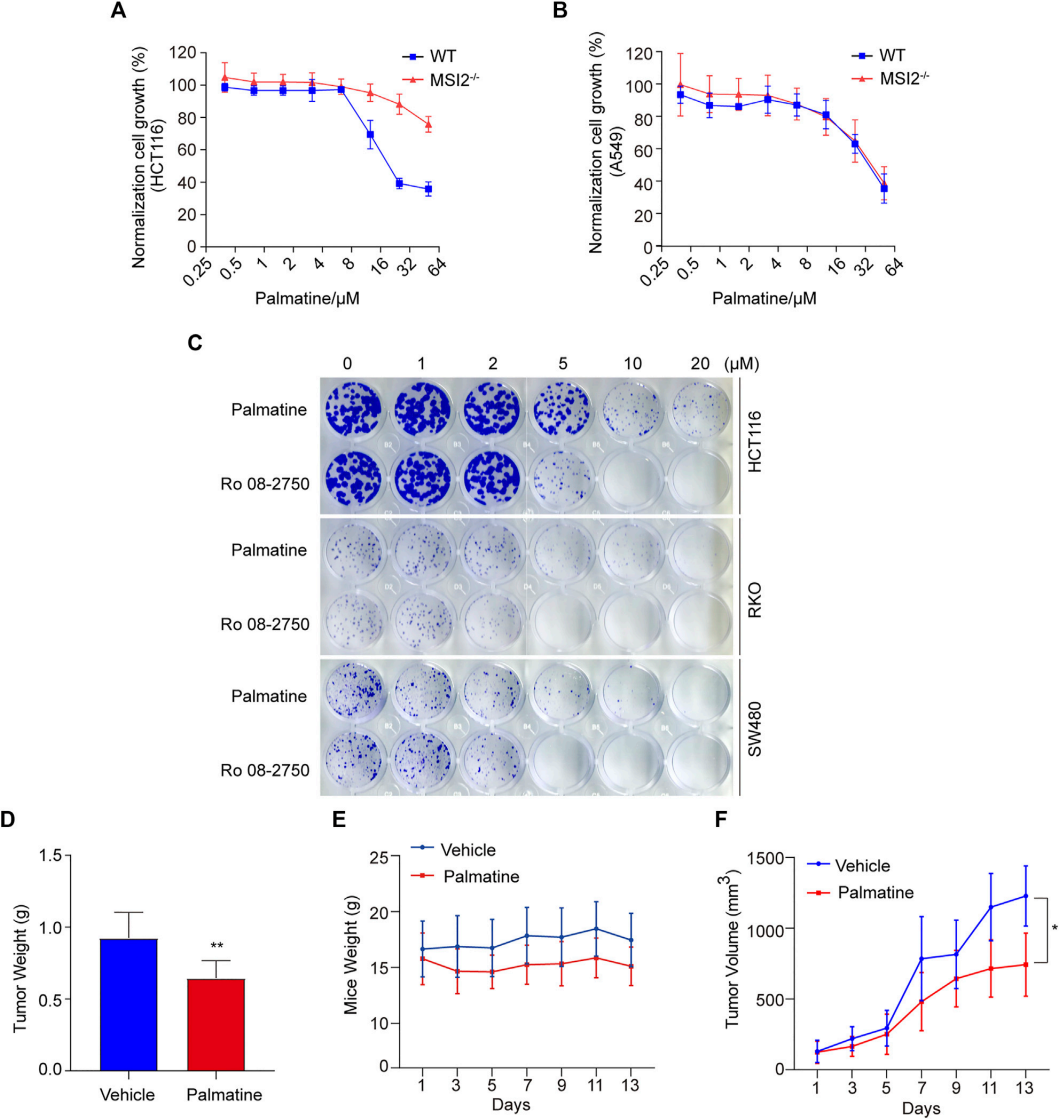
Palmatine inhibited colon cancer *in vitro* and *in vivo*. **(A**,**B)** CCK8 assay of WT (blue) and MSI2^−/−^ (red) HCT116 **(A)** or A549 **(B)** cells upon treatment of palmatine for 72 h at indicated doses. Quantifications of three independent experiments were represented as means ± SD. **(C)** Colony formation of HCT116, RKO, and SW480 cells. Cells were treated with palmatine or Ro 08-2750 with indicated concentrations for 8 days before staining with crystal violet; colony formation was quantified by OD 590 nm measurement and was normalized to the WT. **(D)** HCT116 cells xenograft tumors’ weight treated with palmatine as indicated for 13 days. *p* < 0.01, unpaired *t*-test (two tailed). **(E)** Mice weight of HCT116 cells xenograft nude mice treated with palmatine as indicated for 13 days. **(F)** Growth curves of HCT116 cells xenograft tumors generated by palmatine treatment in nude mice. *p* < 0.05, two-way ANOVA. Data were presented as mean ± SD of six mice in each group.

Palmatine is an isoquinoline alkaloid that showed anti-cancer activity through anti-proliferation, cell cycle arresting, and inducing apoptosis in a variety of cancers such as colorectal and prostate cancers (Hambright et al., 2015; Liu et al., 2020). To further characterize the MSI2-dependent cancer cell inhibition of palmatine, we firstly examined palmatine in a dose-course assay, and confirmed the different responses between wild-type and MSI2 knockout HCT116 cells (Figure 5A), although palmatine exerted a weaker inhibition than Ro 08-2750 on the proliferation of colon cancer HCT116, RKO, and SW480 cells (Figure 5C). Consistent with unchanged growth of A549 cells upon MSI2 depletion (Figures 1J,K), no significant difference of growth inhibition was observed between the wild-type and MSI2 knockout A549 cells upon palmatine treatment (Figure 5B). The anti-cancer effect of palmatine was assessed using the HCT116 xenograft tumor model. Of note, although xenograft tumor growth was significantly reduced by treatment with palmatine at 50 mg/kg for 13 consecutive days (Figure 5D), 20% weight loss was observed in the palmatine group from the third day (Figure 5E). Interestingly, this percentage of weight loss was maintained until the 13th day (Figure 5E). It seemed that the first 3 days’ treatment of palmatine caused a toxic effect on mice, whereas the mice were tolerated and the weight developed similar to that of the vehicle in the following treatment (Figure 5E). Of note is that the tumor growth was significantly slower compared to the vehicles since 7 days of drug administration (Figure 5F), indicating the anti-tumor effect of palmatine besides its toxicity at 50 mg/kg/day. Nevertheless, the safe dose of palmatine to the xenograft mice model needs further clarification in the future.

## Discussion

Drugging RBPs has been considered challenging since many of them do not have a typical pocket for binding and inhibition. Although previous studies reported 18–22 carbon ω-9 monounsaturated fatty acids and (−)gossypol inhibit the RNA-binding activity (Lan et al., 2018) of MSI, the selectivity and requirement of MSI to the two compounds are to be clarified (Clingman et al., 2014; Lan et al., 2015; Lan et al., 2018). A later study identified Ro 08-2750 as a selective MSI inhibitor based on its RNA-binding inhibition of both MSI1 and MSI2 (Minuesa et al., 2019). However, the depletion of MSI2 did not attenuate the cytotoxicity of both (−)gossypol and Ro 08-2750 (Figures 3A,B), indicating that there are other functional targets of the compounds in colon cancer cells.

Inspired by the “golden standard” in evaluating a drug target, we developed a direct strategy to identify MSI2 inhibitors based on the loss–rescue phenotype assay, in that MSI2 is required for the phenotypes of candidate inhibitors. Here, we reported palmatine as an MSI2-dependent inhibitor, and the loss of MSI2 significantly rescued the inhibition of palmatine to HCT116 cells. Notably, the direct engagement of palmatine and MSI2 was confirmed *in vitro* and *in vivo* assays. It is important to note that palmatine is also bound to the MSI family member MSI1 and other RBPs, and the affinities were comparable in both MSI proteins. Further structure–activity relationship study is worthy to elevate the efficiency and selectivity in the future work.

Another important finding is the candidate target mRNAs of MSI2 in colorectal cancer. In the present study, we developed strategies to identify MSI2 targets and inhibitors using the MSI2 knockout cells. Through the profiling of gene expression upon the depletion of MSI2, we identified 38 genes in colorectal cancer cells with altered protein expression versus the unchanged RNA level; all of them were specifically regulated by MSI2 in the context of colon cancer but not NSCLC cells. In particular, KIF18A, a member of the kinesin-8 family, has been shown to be overexpressed in malignant tumors like colon, breast, lung, pancreas, prostate, cervix, and ovarian cancers (Zhang et al., 2010). Importantly, the dysregulation or deletion of KIF18A results in longer spindles and aberrant chromosome segregation, which contribute to the initial onset of cancer (Wilmeth et al., 2010). Another target EIF4EBP1, also known as 4EBP1, encodes a translation repressor protein (Gingras et al., 1999). The overexpression of EIF4EBP1 is observed in multiple cancers such as hepatocellular and breast cancers and associated with poor prognosis of the patients (Karlsson et al., 2011; Karlsson et al., 2013; Cha et al., 2015). The functional link between MSI2 and these targets gives us a better idea about the role of MSI2 in tumorigenesis. However, most of the reported MSI2 targets exhibited an unexpected expression pattern under our analysis, indicating the specific RNA targets in colon cancer cells and the limitation of our strategy.

In conclusion, we developed MSI2 knockout cancer cells and demonstrated that MSI2 is required for the survival of colorectal cancer HCT116 cells but not NSCLC A549 cells. A global profiling of the transcriptome and proteomic of MSI2 knockout colorectal cells revealed 38 candidate MSI2-targeted genes. Furthermore, in a loss–rescue screening based on the MSI2 knockout cancer cells, palmatine was identified as a functional MSI2 antagonist inhibiting the MSI2-dependent growth of colorectal cancer cells. We also confirmed that palmatine is directly bound to MSI2 at its C-terminal. Our findings not only indicated MSI2 as a promising therapeutic target of colorectal cancer but also provided a small molecule palmatine as a direct and functional MSI2 antagonist for cancer therapy.

## Data Availability

The data presented in the study were deposited in the Sequence Read Archive (SRA) repository (https://www.ncbi.nlm.nih.gov/sra), accession number SRR16831052, SRR16831053, SRR16831054, and SRR16831055. All the above data has been released.
